# Detection of *Anopheles stephensi* Mosquitoes by Molecular Surveillance, Kenya

**DOI:** 10.3201/eid2912.230637

**Published:** 2023-12

**Authors:** Eric O. Ochomo, Sylvia Milanoi, Bernard Abong’o, Brenda Onyango, Margaret Muchoki, Diana Omoke, Evelyn Olanga, Laban Njoroge, Elijah Omondi Juma, James Dan Otieno, Damaris Matoke-Muhia, Luna Kamau, Cristina Rafferty, John E. Gimnig, Mildred Shieshia, Daniel Wacira, Joseph Mwangangi, Marta Maia, Charles Chege, Ahmeddin Omar, Martin K. Rono, Lucy Abel, Wendy Prudhomme O’Meara, Andrew Obala, Charles Mbogo, Lenson Kariuki

**Affiliations:** Kenya Medical Research Institute, Nairobi, Kenya (E.O. Ochomo, S. Milanoi, B. Abong’o, B. Onyango, M. Muchoki, D. Omoke, D. Matoke-Muhia, L. Kamau, J. Mwangangi, M. Maia, M.K. Rono, C. Mbogo);; PMI Kinga Malaria Project, Abt Associates Inc., Kisumu, Kenya (E. Olanga);; National Museums of Kenya, Nairobi (L. Njoroge);; Pan-African Mosquito Control Association, Nairobi (E.O. Juma, D. Matoke-Muhia, C. Mbogo);; World Health Organization, Kenya Country Office, Nairobi (J.D. Otieno);; Centers for Disease Control and Prevention, Atlanta, Georgia, USA (C. Rafferty, J.E. Gimnig);; US President’s Malaria Initiative, Washington, DC, USA (C. Rafferty, J.E. Gimnig, M. Shieshia, D. Wacira);; University of Oxford, Oxford, UK (M. Maia);; KEMRI-Wellcome Trust, Kilifi, Kenya (M. Maia, M.K. Rono);; Division of National Malaria Program, Ministry of Health, Nairobi (C. Chege, A. Omar);; Moi University, Eldoret (W.P. O’Meara, A. Obala); AMPATH, Eldoret, Kenya (L. Abel);; Duke University, Durham, North Carolina, USA (W.P. O’Meara, L. Kariuki)

**Keywords:** malaria, *Anopheles stephensi*, vector-borne infections, parasites, mosquitoes, molecular methods, Marsabit, Turkana, Kenya

## Abstract

The *Anopheles stephensi* mosquito is an invasive malaria vector recently reported in Djibouti, Ethiopia, Sudan, Somalia, Nigeria, and Ghana. The World Health Organization has called on countries in Africa to increase surveillance efforts to detect and report this vector and institute appropriate and effective control mechanisms. In Kenya, the Division of National Malaria Program conducted entomological surveillance in counties at risk for *An. stephensi* mosquito invasion. In addition, the Kenya Medical Research Institute conducted molecular surveillance of all sampled *Anopheles* mosquitoes from other studies to identify *An. stephensi* mosquitoes. We report the detection and confirmation of *An. stephensi* mosquitoes in Marsabit and Turkana Counties by using endpoint PCR and morphological and sequence identification. We demonstrate the urgent need for intensified entomological surveillance in all areas at risk for *An. stephensi* mosquito invasion, to clarify its occurrence and distribution and develop tailored approaches to prevent further spread.

The *Anopheles stephensi* mosquito is a major vector of malaria in south Asia, the Middle East, and southern China, where it is endemic and is known to transmit both *Plasmodium falciparum* and *P. vivax*. This mosquito differs from other malaria vectors because of its ability to grow and reproduce in human-made containers in clean or contaminated water. Those traits have enabled *An. stephensi* mosquitoes to colonize urban settings, in addition to their native rural foci, where they can potentially sustain malaria transmission (*1*). 

The *An. stephensi* mosquito was first reported in Djibouti in the Horn of Africa in 2012 (*2*). Since then, it has been reported in multiple urban and rural settings in Ethiopia, Sudan, Somalia, and Ghana (*3*–*6*) and could be responsible for sustaining malaria transmission in Ethiopia. The species has the potential to increase *P. falciparum* incidence by 50% according to recent mathematical modeling (*7*,*8*), as has been observed in Djibouti (*9*).

*An. stephensi* mosquitoes could spread south and west from their original foci of detection in the Horn of Africa, as has been observed in Nigeria (*4*) and Ghana (*6*). This vector has the potential to establish or increase transmission in urban settings where the malaria burden is generally lower than in rural settings, particularly in areas where poorly planned drainage and waste disposal systems create conducive larval habitats (*10*). The behavior of adult mosquitoes in their invasive range in Africa is not well understood, especially as they continue to colonize new areas in the continent, but their spread has been predicted using modeling (*10*). 

The World Health Organization recently called for heightened surveillance and development of response strategies to limit the spread of this vector in Africa (*4*). The initiative highlights 5 key focus areas: increased collaboration across sectors and borders, strengthening surveillance, improving information exchange, developing national guidelines, and prioritizing research to evaluate tools against this vector. In Kenya, the Division of National Malaria Program (DNMP) at the Ministry of Health and its partners have been on high alert and instituted surveillance efforts after the World Health Organization initiative (*4*). Surveillance efforts have been focused along the Kenya coast and the northern counties bordering Sudan and Ethiopia. Current surveillance efforts are aimed at the collection of both larval and adult mosquito samples. Samples collected are identified using morphological keys and PCR at the reference laboratories located at the Kenya Medical Research Institute (KEMRI). Here we detail the process that led to detecting and identifying *An. stephensi* mosquitoes in Kenya.

## Methods

### Surveillance Sites

**Figure 1:**
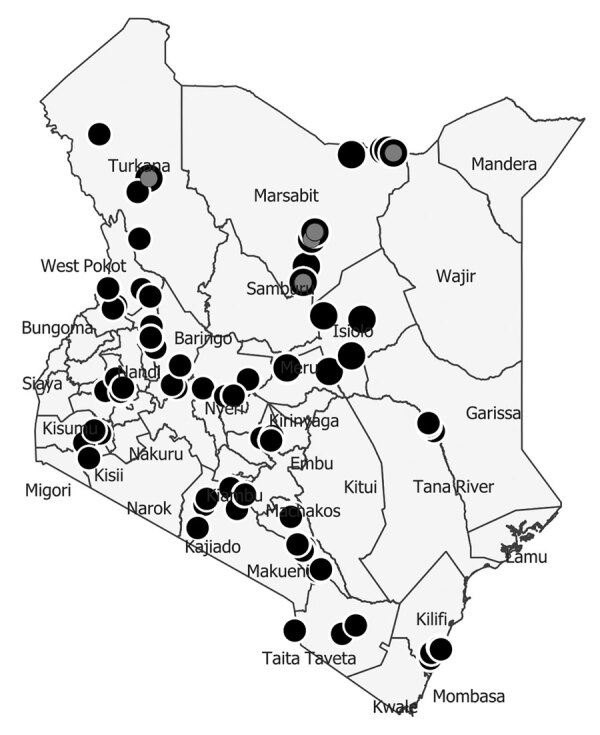
Locations of mosquito collection during surveillance conducted by Division of National Malaria Program and partners, Kenya, December 2022. Gray filled circles indicate sites where *Anopheles stephensi* mosquitoes were present; black filled circles indicate sites where only other vectors (*An. gambiae* and *An. funestus* mosquitoes) were collected.

The DNMP and its partners collected mosquitoes in 14 counties in December 2022 as part of routine surveillance. Counties where DNMP supported vector surveillance in December 2022 were categorized as malaria endemic (Kilifi, Taita, and Taveta), highland epidemic prone (Elgeyo Marakwet, West Pokot, Kisii, and Nandi), low risk (Garissa, Makueni, Kajiado, Kirinyaga, and Laikipia), or seasonal (Marsabit, Baringo and Turkana) (Figure 1). For the purpose of this work, we present results for Marsabit and Turkana Counties, where samples were collected, identified, and confirmed to be *An. stephensi* mosquitoes. Marsabit and Turkana are neighboring counties in northern Kenya, located on either side of Lake Turkana. Marsabit County borders Ethiopia to the north, Turkana County to the west, Samburu County to the south, and Wajir and Isiolo Counties to the east. Turkana County borders Uganda to the west, South Sudan to the north, and Ethiopia to the northeast. Directly east lies Lake Turkana, and Marsabit lies just beyond. The counties lie 300–900 meters above sea level. 

Mosquito sampling in Marsabit was conducted in the subcounties of Moyale, Laisamis, and Saku and focused on urban and rural settings along the northern transport corridor connecting Kenya and Ethiopia (Table 1). Sampling in Turkana focused on Lodwar, the capital of the county and a major town on the land transport corridor into Kenya. The main economic activities of the rural population are nomadic pastoralism because of the semiarid terrain; urban trade centers are set up along the northern transport corridor. Urban trade centers were the focus of the sampling efforts.

**Table 1:** Characteristics of the habitats from which *Anopheles* spp. mosquito larvae were collected, Kenya*

County	Subcounty	Locality village	Habitat type	Larvae presence	Latitude	Longitude	Elevation, m
Marsabit	Saku	Mountain	Tank	Yes (*An. stephensi*)	2.329833	37.996728	1,319.14
Marsabit	Saku	Nagayo	Tire	Yes (*An. stephensi*)	2.337666	37.990045	1,353.7
Marsabit	Saku	Karare	Streambed	No	2.227091	37.938076	1,057.33
Marsabit	Saku	Saku Central	Water treatment plant	Yes (*An. stephensi*)	2.322261	37.982904	1,437.76
Marsabit	Laisamis	Malgis	Riverbed	Yes	1.832333	37.86083	453.76
Marsabit	Laisamis	Laisamis	Animal drinking points	Yes (*An. stephensi*)	1.587676	37.80819	541.84
Turkana	Turkana Central	Nakwamekwi	River pan (3 sites)	Yes (*An. stephensi*)	3.110770	35.573982	NA
Turkana	Turkana Central	St. Monicah	Drainage ditch (2 sites)	Yes	3.110497	35.574072	NA
Turkana	Turkana Central	St. Monicah	Cisterns (2 sites)	Yes	3.110497	35.574072	NA
Turkana	Turkana Central	Kanamkemer	Cisterns (3 sites)	Yes	3.106836	35.604599	NA
Turkana	Turkana Central	Natot	Irrigation canal (1 site)	Yes	3.108565	35.579132	NA

*NA, not available.

### Sampling

**Figure 2:**
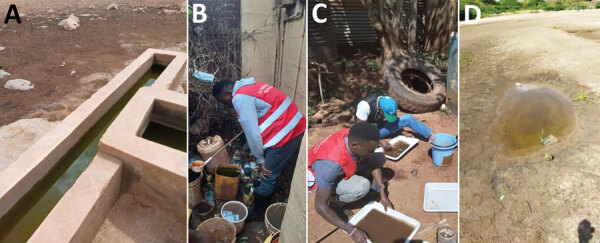
Habitats where *Anopheles stephensi* mosquitoes were collected, Kenya. A) Animal watering pan in Marsabit County; B) disposed containers containing standing water in Marsabit County; C) old tire in Marsabit County; D) seasonal river pan in Turkana County. Persons pictured gave consent for their photographs to be published in this article.

We conducted mosquito sampling in Marsabit for adult and larval samples. We collected adult mosquitoes using US Centers for Disease Control and Prevention (CDC) light traps and collected larvae by dipping. We set CDC light traps overnight indoors, next to a person sleeping under a bednet, or outside, 10 m from the structure without regard for the presence of animals, between 6 pm and 7 pm and collected them the next morning between 7 am and 8 am. In addition, we dipped for larvae in animal watering pens, containers, tires, and other standing water in the area (Figure 2). We collected *Anopheles* larvae and placed them in whirlpacks for transportation to the entomology laboratory at KEMRI for additional assays. The mosquitoes were reared in the infection room; the room was equipped with a triple door and curtains at the entrance and sealed windows to prevent escapees. Surviving larvae were reared to adults for morphologic identification using standard conditions (25 + 2°C; 80% + 4% relative humidity; 12 h/12 h light/dark cycle). We fed larvae on Tetramin baby fish food and brewer’s yeast daily and maintained adults on 10% sugar solution.

In Turkana, larval sampling focused on water pans near the seasonal river and cement water cisterns. We visited 11 suspected larval sites throughout the town every 2 weeks and dipped 5 times at each site to quantify larval density. We separated *Anopheles* larvae and placed them in tubes with 95% ethanol for shipment to the PEARL laboratory in Webuye. Collections occurred during November–December 2022.

### Molecular Characterization

We isolated DNA from 55 mosquito carcasses (either whole or legs and wings) consisting of field-collected larvae or laboratory-reared F_0_ adults from larvae collected in Marsabit using the ethanol precipitation method (*11*) in the KEMRI Kisumu laboratory. We conducted amplification using an endpoint PCR assay that included 3 primers: St-F (5′-CGTATCTTTCCTCGCATCCA-3′), an *An. stephensi*–specific forward primer targeting the internal transcribed spacer 2 (ITS2*)* region; U5.8S-F (5′-ATCACTCGGCTCATGGATCG-3′). a universal forward primer flanking the conserved 5.8S rDNA region; and UD2-R (5′-GCACTATCAAGCAACACGACT-3′), a universal reverse primer flanking the conserved D2 domain of 28S rDNA (*12*). We performed the reactions using 0.15 µL of the DNA template alongside a positive control with the following set of cycling conditions: 95°C for 5 min, followed by 95 °C for 30 s, 55 °C for 30 s and 68 °C for 45 s for 30 cycles, and a final extension at 68 °C for 7 min. Thereafter, we ran 15 µL of each of the PCR products on 2% agarose gel alongside 3 µL of a 100-bp DNA ladder for size comparison. We visualized the products in the gel documentation system for an expected amplicon size of ≈438bp. This visualization was the primary method of identification given the relative inexperience of the laboratory teams in morphological identification of *An. stephensi* mosquitoes*.*

Samples collected in Turkana were processed at the AMPATH Laboratories in Eldoret, Kenya. We rinsed field-collected larval samples preserved in ethanol with nuclease-free water and pooled in groups of 3 from the same breeding site. We extracted triads in a single well of a 96-well plate using the Hotshot protocol, performed amplification using a previously published protocol (*13*) with corrected primer sequences, and visualized reactions on 2% agarose gels. If a band of the expected size was observed, we separated the larvae in the pool and extracted them individually using the DNeasy Blood and Tissue Kit (QIAGEN, https://www.qiagen.com), after which we repeated amplification and electrophoresis as previously described. We subsequently sequenced positive samples as described in the following section.

### Morphologic Identification and Sequencing

**Figure 3:**
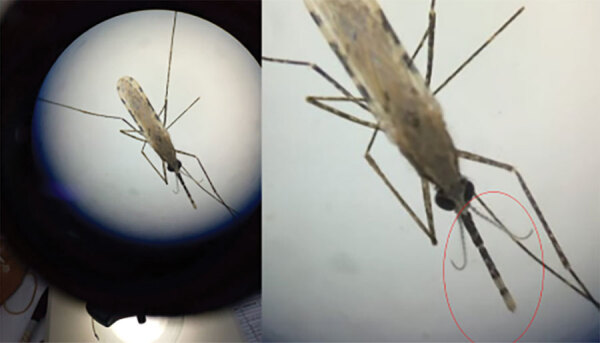
Pictures of *Anopheles stephensi* mosquito collected in Kenya as seen under a microscope. The dual banding on the palps characteristic of *An. stephensi* mosquitoes is circled in red in the closer image at right. Other distinguishing features are not clear in this image.

We taxonomically identified emerging adults that were a subset of the larvae collected in Marsabit using the keys described by Coetzee et al. (*14*) to detect the distinct banding on the maxillary palps, pale scales on the scutum, and the 3 dark spots on wing vein 1A (Figure 3). We randomly selected 4 adult specimens that were a subset of the samples from Marsabit identified as *An. stephensi* mosquitoes by morphology and shipped them to CDC (Atlanta, GA, USA), where DNA from a single mosquito leg was extracted using the Extracta DNA Prep for PCR kit (Quantabio, https://www.quantabio.com). We performed amplification targeting the ITS2 (as previously described) and the cytochrome c oxidase subunit 1 gene (CO1) locus. For CO1 amplification, we used specific LCO1490F (5ʹ-GTTCAACAAATCATAAAGATATTGG-3ʹ) and HCO2198R (5ʹ-TAAACTTCAGGTGACCAAAAAATCA-3ʹ) primers (*15*). The PCR cycling conditions included an initial step at 95°C for 1 min, then 30 cycles of denaturation at 95°C for 30 s, annealing at 48°C for 30 s, and extension at 72°C for 1 min. We ran Amplicons for both ITS2 and CO1 on a 2% agarose for confirmation, then used the positive PCR products for Sanger sequencing. We performed BLAST (https://blast.ncbi.nlm.nih.gov/Blast.cgi) homology searches of both ITS2 and CO1 sequences using the default parameters to confirm the matching species.

We sequenced 4 larval samples from Lodwar after the ITS2 band was purified from the agarose gel at the KEMRI Wellcome Trust (Kilifi, Kenya). We constructed sequencing libraries using Oxford Nanopore Technologies Ligation Sequencing Kit and multiplexed samples using the Native Barcoding Expansion Kit (https://nanoporetech.com). We performed adaptor ligation on the barcoded amplicon pool and the final library loaded on a SpotON R9.4.1 flow cell and sequenced on the GridION (Oxford Nanopore Technologies).

Using SPADES assembler (*16*), we performed de novo assembly on the filtered reads. We performed species identification through a BLAST search using ITS2 sequences from GenBank as the subject database and the assembled contigs as the query dataset.

### Phylogenetic Analyses

We constructed phylogenetic trees for both CO1 and ITS2 sequences by incorporating sequences from diverse isolates retrieved from GenBank along with isolates from Kenya. We used MAFFT software version 7.520 (*17*,*18*) for all sequence alignments and reconstructed maximum-likelihood phylogenies using the Bayesian Information Criterion with general time-reversible (GTR) as the best substitution model as inferred by jModelTest in IQ-TREE version 2.0.7 (*19*,*20*). We performed tree visualization using MEGA version 11 (*21*) and took the bootstrap consensus tree inferred from 1,000 replicates to reliably show the evolutionary history.

## Results

### Molecular Surveillance Results

We collected *Anopheles* larvae from 11 locations in 3 subcounties in the 2 counties (Table 1). In Marsabit, a total of 59 larvae were collected. Of those, 11 died in transit and were immediately prepared for PCR identification using the *An. stephensi* protocol; 7 were confirmed as *An. stephensi* mosquitoes (Table 2). We pooled the 48 remaining larvae by subcounty to rear adult samples. Of the first 12 samples that emerged, we identified 9 adults by morphology (Figure 3). We correctly identified 7 of the 9 samples as *An. stephensi* mosquitoes, which were later confirmed by PCR through ITS2 amplification (Table 2). The other 2 were identified as *An. gambiae* mosquitoes by morphology but were confirmed to be *An. stephensi* mosquitoes by PCR. We shipped 4 of those samples to the CDC for sequencing as described previously; 36 samples did not amplify using *An. stephensi*, *An. gambiae*, or *An. funestus* PCR protocols and are the subject of further investigation. We did not conduct morphologic identification on those samples before DNA extraction; the samples will be sequenced to determine species at a later date. No adult mosquitoes were collected in light traps. In summary, of the 59 mosquito samples tested by PCR from collections in Marsabit, 23 were confirmed to be *An. stephensi* mosquitoes.

**Table 2:** Numbers of mosquito larvae or adults used for PCR to identify *Anopheles stephensi* mosquitoes, Kenya*

Subcounty	Total *Anopheles* larvae collected	Larvae used for PCR				Adults used for PCR		
		Sample size	*An. stephensi*	Unamplified		Sample size	*An. stephensi*	Unamplified
Saku	42	9	6	3		33	8	25
Laisamis	17	2	1	1		15	8	7
Moyale	51	51	2	27		NA	NA	NA
Turkana Central†	193	193	5	188		NA	NA	NA

*Unamplified samples are those that failed to amplify with the *An. stephensi*, *An. gambiae*, and *An. funestus* PCR protocols. NA, not available. †Including only a single site that was positive for *An. stephensi*. Five larvae were shipped to KEMRI-Wellcome Trust for sequencing.

Of the 9 sites monitored in Lodwar town during November 8–December 22, 2022, two had only culicine larvae and 7 had *Anopheles* larvae. A total of 1,415 larvae were collected and screened by PCR; 1,218 were collected from river pans, 50 from cisterns, 147 from drainage ditches, and the remaining from other sources. Two pooled extracts from river pans on the Turkwel River screened positive for *An. stephensi.* We separated, extracted, and retested 5 larvae; 5 were confirmed to be *An. stephensi* mosquitoes*.*

### Sequencing

**Figure 4:**
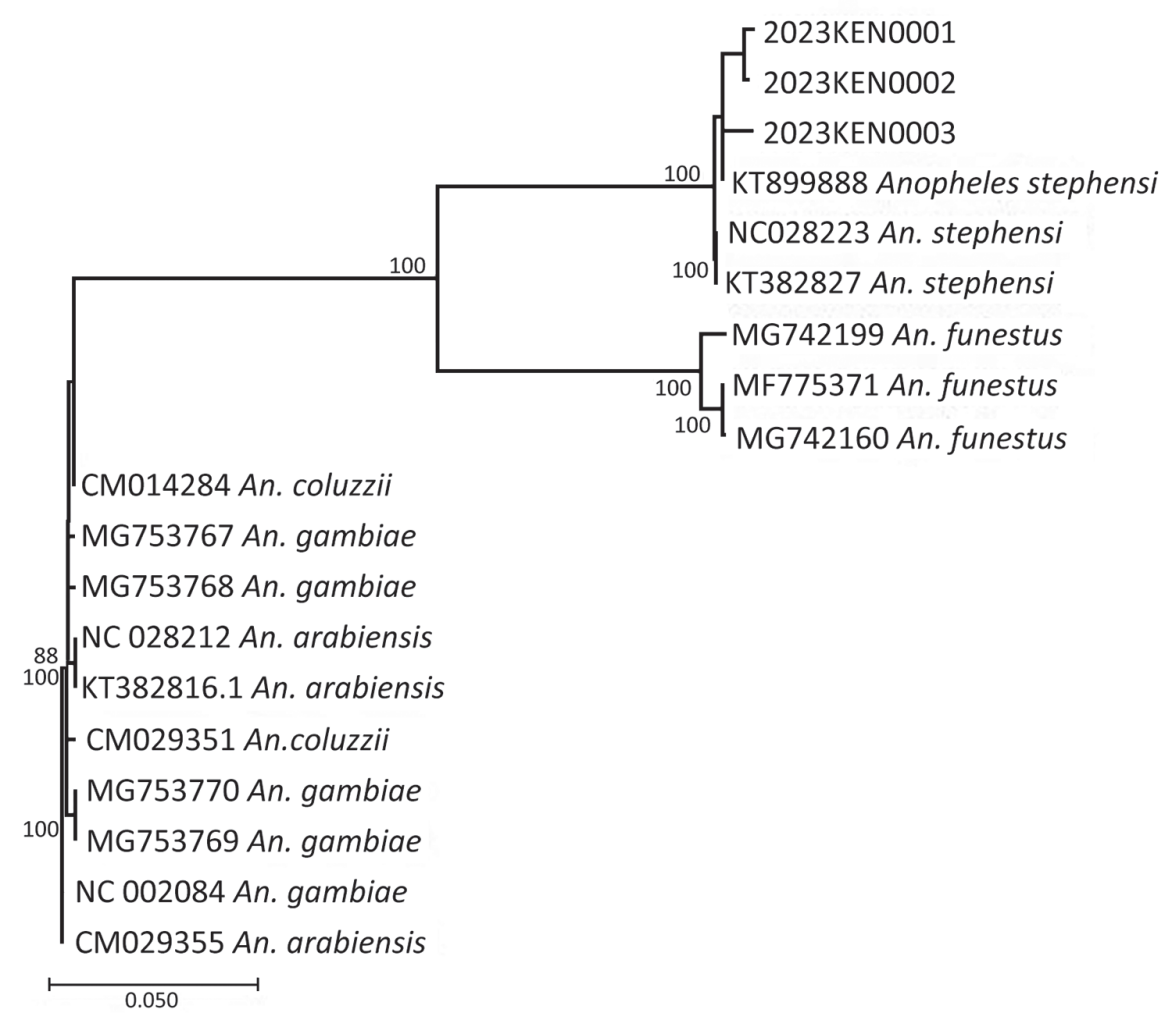
Phylogenetic tree representing the relationship of *Anopheles stephensi* mosquito isolates from Kenya (2023KEN0001, 2023KEN0002, and 2023KEN0003) and reference *Anopheles* spp. isolates using the cytochrome c oxidase subunit 1 region. GenBank accession numbers are provided for reference sequences; accession numbers for Kenya sequences are provided in Table 3. Scale bar indicates 5% nucleotide sequence divergence. Values on the branches represent the percentage of 1,000 bootstrap replicates; bootstrap values >70% are shown in the tree.

The sequences for 3 of the 4 adult samples matched CO1 isolates from GenBank and were confirmed as *An. stephensi* mosquitoes (Figure 4)*.* One sample failed to amplify, possibly because of DNA degradation (Table 3). BLAST searches using default parameters for isolates 2 and 3 matched to *An. stephensi* sequences with 100% identity to the hap 10 5.8S ribosomal RNA gene, ITS2; isolate 1 had 99.4% identity to the same gene but 100% identity to the *An. stephensi* isolate 141 steph 5.8S ribosomal RNA gene, ITS2. However, when we focused on the CO1 genes in BLAST searches, we found that isolates 1 and 2 exhibited a striking similarity of 100% (isolate 1) and 99.7% (isolate 2) to *An. stephensi* isolate SM147. Conversely, isolate 3 displayed a substantial 99% identity to *An. stephensi* isolate ANST15 (Table 3).

**Table 3:** Results for sequencing analysis of 4 mosquito samples from Marsabit County, Kenya, sent to CDC for identification in study of *Anopheles stephensi* mosquito, Kenya

Origin sample ID	CDC sample ID	Morphologic ID	Confirmed species	ITS2 sequences				CO1 sequences		
				GenBank accession no.	GenBank accession no. of closest match	% Identity match		GenBank accession no.	GenBank accession no. of closest match	% Identity match
KE83GY	2023KEN0001	Suspected *An. stephensi*	*An. stephensi*	OQ275144	FJ526599.1	99.40		OR607949	OM801708	100
KE83QF	2023KEN0002	Suspected *An. stephensi*	*An. stephensi*	OQ275145	MW732931.1	100		OR607950	OM801708	99.70
KE83FZ	2023KEN0003	Suspected *An. stephensi*	*An. stephensi*	OQ275146	MW732931.1	100		OR607951	MN329060.1	99.10
KE831Q	2023KEN0004	Suspected *An. stephensi*	Did not amplify		NA	NA			NA	NA

*CDC, US Centers for Disease Control and Prevention; CO1, cytochrome c oxidase subunit 1; ID, identification; ITS2, internal transcribed spacer 2.

**Figure 5:**
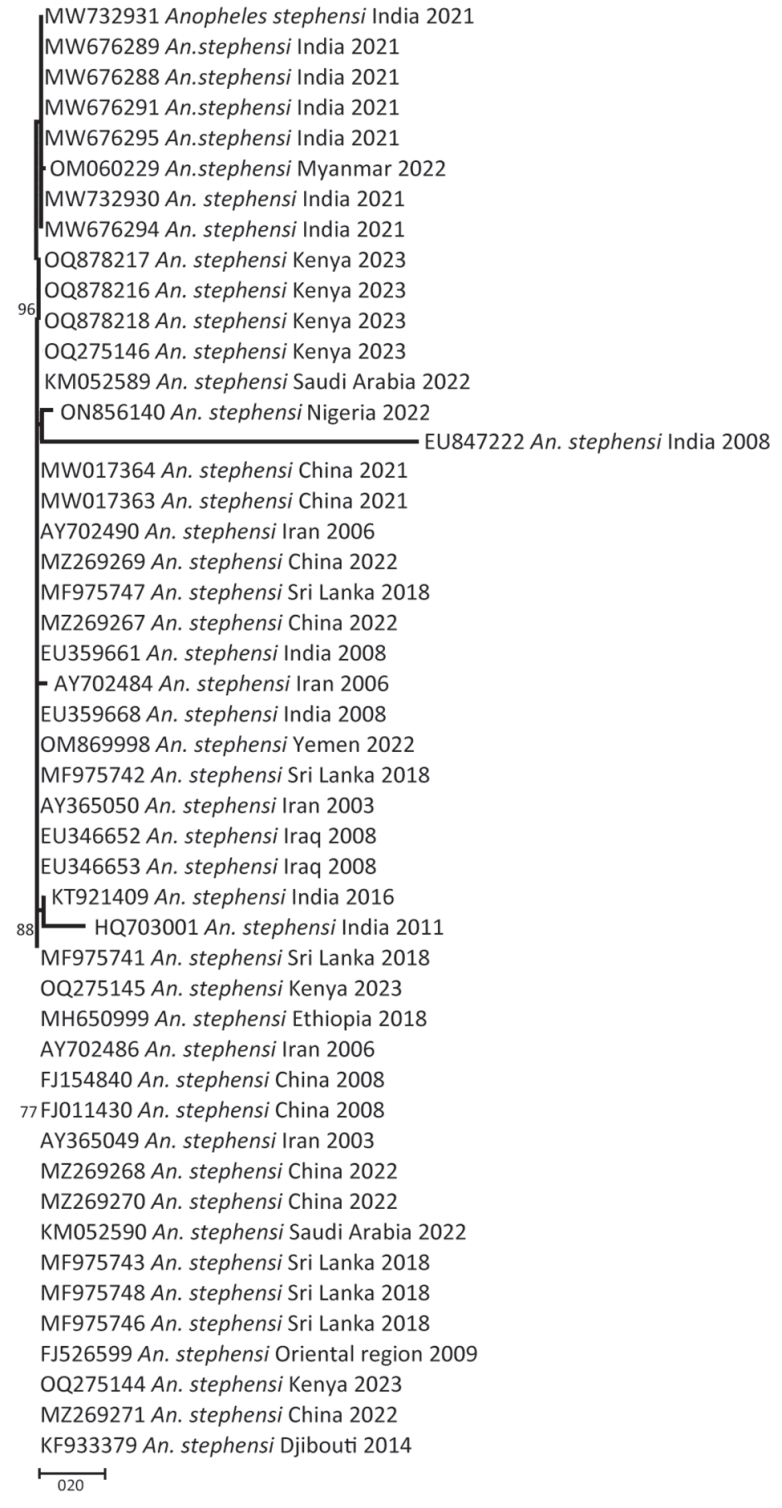
Phylogeny of cytochrome c oxidase subunit 1 sequences of *Anopheles stephensi* mosquito isolates from Kenya (2023KEN0001, 2023KEN0002, and 2023KEN0003) and reference *An. stephensi* mosquito isolates retrieved from GenBank. GenBank accession numbers are provided for reference sequences; accession numbers for Kenya sequences are provided in Table 3. Scale bar indicates 1% nucleotide sequence divergence. Values on the branches represent the percentage of 1,000 bootstrap replicates; bootstrap values >70% are shown in the tree.

**Figure 6:**
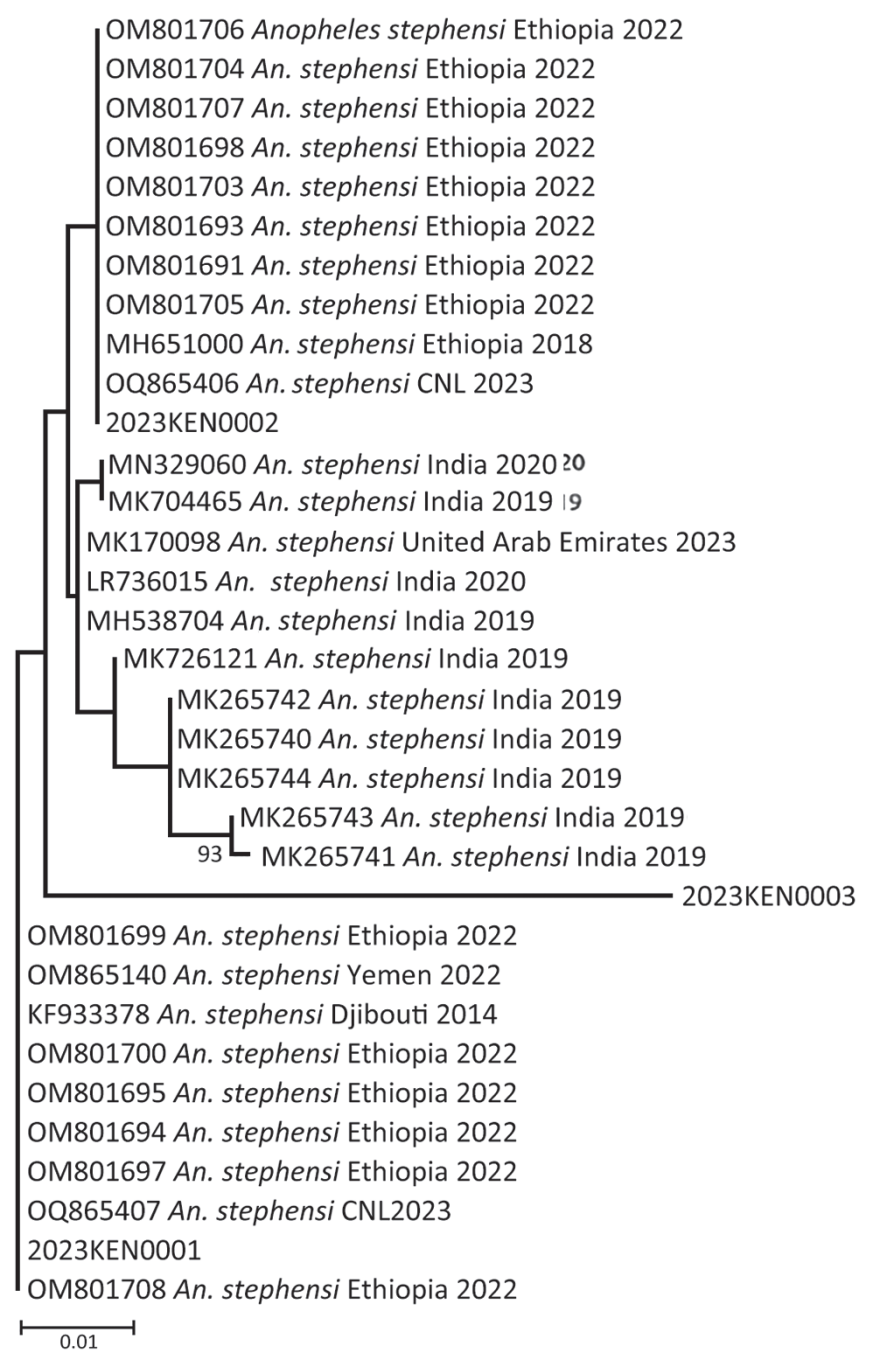
Phylogeny of internal transcribed spacer 2 sequences of *An. stephensi* isolates from Kenya (OQ275146.1, OQ275144.1, OQ275145.1, OQ878216, OQ87821, and OQ878218) in comparison to *An. stephensi* isolates from other parts of the world. GenBank accession numbers are provided for reference sequences; accession numbers for Kenya sequences are provided in Table 3. Scale bar indicates 20% nucleotide sequence divergence. Values on the branches represent the percentage of 1,000 bootstrap replicates; bootstrap values >70% are shown in the tree.

In-depth phylogenetic analyses of the CO1 sequences from Kenya isolates 1 and 2 demonstrated a close relationship with sequences from Ethiopia isolates; isolate 3 exhibited a close association with sequences from India (Figure 5). On the other hand, phylogenetic analysis of sequenced isolates with other isolates of ITS2 for *An. stephensi* available in GenBank demonstrated that the isolates from Marsabit and Turkana matched quite closely; however, because ITS2 is a nuclear marker, it was not used to infer relatedness. The isolates closely matched the Iraq, India, Yemen, and Nigeria isolates (Figure 6). The *An. stephensi* sequences from this study have been uploaded to GenBank (accession nos. OQ275144, OQ275145, and OQ275146 [ITS2 sequences from Marsabit]; OQ878216, OQ878217, and OQ878218 [sequences from Turkana]; and OR607949, OR607950, and OR607951 [CO1 sequences from Marsabit]).

## Discussion

We report collection and detection of *An. stephensi* mosquitoes from Marsabit and Turkana Counties in northern Kenya. From the samples collected, we used multiple methods for identification, including morphologic keys, standard ITS2 and CO1 PCR, and Sanger and next-generation sequencing. Molecular methods were instrumental in confirming the presence of *An. stephensi* mosquitoes. The mosquitoes were collected as larvae. The lack of adult mosquitoes found in the light traps indicates the need for studies to characterize adult vector bionomics and behavior to elucidate how they contribute to transmitting malaria and to design appropriate tools for surveillance of adult *An. stephensi* mosquitoes.

Reports from other sites have documented the difficulty of trapping adult mosquitoes (*7*). The bionomics and behavior of this vector in its recent invasive geographic foci are poorly understood; the only available detailed descriptions are from Asia (*4*,*13*,*22*). However, reports from Ethiopia on this vector have indicated that crepuscular biting behaviors and resting outside houses could translate to reduced efficacy of core vector-control interventions, insecticide-treated bed nets, and indoor residual spraying, indicating the importance of accurate parameters (*8*,*13*,*23*). In addition, the effectiveness of any insecticide-based control method will depend on the insecticide resistance of the *An. stephensi* mosquito; insecticide-resistance surveys are needed (*8*,*23*).

On the basis of the phylogenetic analysis of ITS2, the Kenya *An. stephensi* isolates from Turkana and Marsabit matched one another closely, but because we only conducted CO1 analysis of mosquitoes collected from Marsabit, data were insufficient to infer relatedness. The isolates also matched closely with isolates from India, Iraq, Yemen, Iran, and Nigeria but were more distant from the isolates from Ethiopia. However, phylogenetic analysis of CO1 demonstrated 2 of the Marsabit samples matched closely with isolates from Ethiopia, meaning they are likely related and suggesting a southward invasion of *An. stephensi* mosquitoes from Ethiopia. This finding asserts the importance of sequencing CO1 amplicons to infer common phylogenetic origins of *An. stephensi* species. Additional population genetics studies using whole-genome sequencing approaches to describe these clades are needed, along with intensive surveillance to describe their bionomics and behavior. 

Our findings also suggest potential introduction routes; *An. stephensi* mosquitoes were found along highways connecting Kenya to Ethiopia and South Sudan, highlighting the need for increased surveillance along major transportation routes, ideally targeting such areas as truck stops and resting sites, weighbridges, and borders. Future work should include phylogenetic analysis of CO1 isolates of *An. stephensi* mosquitoes to understand their origin and spread. Further, tracking parasites that cause malaria cases around the areas where *An. stephensi* mosquitoes have been introduced will be key given that the species is an efficient vector of both *P. falciparum* and *P. vivax*.

Because of rapid, often unplanned, urbanizing in Africa, many urban centers have poor refuse disposal and drainage systems that are potential larval habitats of *An. stephensi* mosquitoes. In addition, because of inadequate social amenities in informal urban settlements, most inhabitants rely on water storage containers for domestic use. Such containers can thus become major breeding habitats for *An. stephensi* mosquitoes, further compounding the problem of malaria transmission in urbanized areas (*2*,*10*). A recent report described the role of construction in urban areas in Ethiopia in propagating *An. stephensi* larval breeding through uncovered cisterns, plastic containers, and pits dug out for brick manufacturing (S. Yared et al., unpub. data, https://www.biorxiv.org/content/10.1101/2023.05.23.541906v1). In this study, *An. stephensi* larvae were collected in riverbeds, which is notable because *An. stephensi* mosquitoes are thought to confine themselves to habitats similar to those of *Aedes* spp. mosquitoes. That level of plasticity in colonizing larval habitats demonstrates the potential for this species to invade rural and urban areas alike. In addition, climate change, which creates suitable climatic conditions for mosquito breeding, also means the potential for the spread and establishment of *An. stephensi* mosquitoes in cities in Africa is great. 

When *An. stephensi* mosquitoes were introduced into Djibouti (*2*), the country was at the preelimination stage for malaria but then spiked to nearly 3,000 reported malaria cases in 2013, just 1 year after the mosquito was first reported. In 2019, just 6 years later, Djibouti reported 49,402 malaria cases (*24*). Modeling of the potential effects of *An. stephensi* mosquito establishment in Ethiopia predicts a surge in *P. falciparum* cases by 50% overall if no additional interventions are put in place; areas of lowest transmission (≈0.1%) are forecast to be affected the most (*8*). Similar models need to be conducted in all areas that are newly invaded to predict the spread and effects of the vector and to learn more about the potential effects of additional interventions.

The breeding habitats of *An. stephensi* mosquitoes are similar to those of *Ae. aegypti* mosquitoes, but the resting and biting behavior of adult *An. stephensi* mosquitoes in their invasive range in Africa is less well understood (*1*,*25*). Evidence of outdoor, crepuscular feeding by this species suggests it might be less affected by insecticide-treated bed nets or indoor residual spraying as a vector-control intervention. Furthermore, *An. stephensi* mosquitoes in Ethiopia were reported to be highly resistant to pyrethroids, carbamates, and organophosphates (*13*). Those traits indicate that alternative vector-control measures and non–vector-control measures might be needed to address the threat of this invasive mosquito. As the national malaria control program develops a vector-control strategy, integrated vector management approaches offer advantages because of the potential benefit of targeting additional vectors on the basis of World Health Organization guidance (*26*,*27*), particularly because of the poor understanding of this vector’s behavior when it colonizes new areas. Deploying an integrated approach provides opportunities to target *Ae. aegypti* and *An. stephensi* vectors for surveillance and control using similar interventions, which could optimize resource allocation and use in the resource-limited settings where *An. stephensi* mosquitoes are currently being reported. Managing larval sources, including by applying larvicides, reducing larval sources, and modifying the environment to make it less conducive to productive mosquito aquatic stages, has been pointed out as a potential strategy for targeting *An. stephensi* mosquitoes, given their tendency to breed in human-made containers in urban areas (*3*,*13*,*23*,*28*; S Yared et al., unpub. data). Other potential vector control tools, including those currently under evaluation, include spatial repellents (*29*), attractive targeted sugar baits (*30*), endectocides (*31*), insecticide-treated clothing (*32*), and genetically modified mosquitoes (*33*). Given the mosquito’s outdoor, early-evening biting behaviors, its resistance to multiple insecticides, and the threat it poses to malaria control efforts, these alternative vector-control approaches might be necessary to sustain gains made against malaria over the past 2 decades.

The first limitation of our study is that samples were collected over a short time frame in a limited number of sites; in Turkana County, we conducted 4 collections in 2 months at 9 sites, and in Marsabit County, collections were performed at 6 sites over 2 months. Therefore, the temporal and spatial extent of the *An. stephensi* mosquito is still largely unknown and is likely more widespread than this initial report would suggest. Furthermore, only larval samples of *An. stephensi* mosquitoes could be collected, pointing to gaps in our understanding of adult behavior and optimal adult sampling tools and methods. Collection of other *Anopheles* species was likely lacking because collections occurred in the dry season, which also demonstrates the potential for *An. stephensi* mosquitoes to sustain transmission in dry seasons, as has been predicted elsewhere (*34*). Last, 75% of samples collected in Marsabit could not be amplified by any of the species identification PCR protocols available in the KEMRI laboratory and will be sequenced once the budget is available. Amplifying those samples is a critical first step in combating this emerging threat. Expanding surveillance activities to mitigate the spread of *An. stephensi* mosquitoes will be key, as will learning more about how this invasive vector is related to recent malaria outbreaks in both counties.

In conclusion, we confirm the presence of *An. stephensi* mosquitoes in northern Kenya, which points to the urgent need to reexamine and expand vector surveillance and control efforts to include this vector. This mosquito vector is likely to sustain and possibly increase malaria transmission in northern Kenya and spread further southward to highly populated urban areas and existing malaria-endemic counties, further compounding the problem of malaria control in the country. Our findings emphasize the need for heightened and tailored surveillance to elucidate the scope of this invasive vector’s spread, to initiate research on the bionomics of the vector, and to advise on targeted control using existing interventions, including those currently under trial.
